# Novel High Flux Poly(m-phenylene isophtalamide)/TiO_2_ Membranes for Ultrafiltration with Enhanced Antifouling Performance

**DOI:** 10.3390/polym13162804

**Published:** 2021-08-20

**Authors:** Mariia Dmitrenko, Anna Kuzminova, Andrey Zolotarev, Vladislav Liamin, Tatiana Plisko, Katsiaryna Burts, Alexandr Bildyukevich, Sergey Ermakov, Anastasia Penkova

**Affiliations:** 1 St. Petersburg State University, 7/9 Universitetskaya Nab., 199034 St. Petersburg, Russia; ai.kuzminova@mail.ru (A.K.); andrey.zolotarev@spbu.ru (A.Z.); lyamin.vlad.322@gmail.com (V.L.); s.ermakov@spbu.ru (S.E.); a.penkova@spbu.ru (A.P.); 2 Sirius University of Science and Technology, 1 Olympic Ave, 354340 Sochi, Russia; 3 Institute of Physical Organic Chemistry, National Academy of Sciences of Belarus, 13 Surganov Str., 220072 Minsk, Belarus; plisko.v.tatiana@gmail.com (T.P.); katyaburt@gmail.com (K.B.); uf@ifoch.bas-net.by (A.B.)

**Keywords:** poly(m-phenylene isophthalamide), titanium oxide, ultrafiltration, fouling, cutting fluid, bovine serum albumin

## Abstract

Wide application of ultrafiltration in different industrial fields requires the development of new membranes with tailored properties and good antifouling stability. This study is devoted to the improvement of ultrafiltration properties of poly(m-phenylene isophtalamide) (PA) membranes by modification with titanium oxide (TiO_2_) particles. The introduction of TiO_2_ particles improved membrane separation performance and increased antifouling stability and cleaning ability under UV irradiation. The developed membranes were characterized by scanning electron and atomic force microscopy methods, the measurements of water contact angle, and total porosimetry. The transport properties of the PA and PA/TiO_2_ membranes were tested in ultrafiltration of industrially important feeds: coolant lubricant (cutting fluid) emulsion (5 wt.% in water) and bovine serum albumin (BSA) solution (0.5 wt.%). The PA/TiO_2_ (0.3 wt.%) membrane was found to possess optimal transport characteristics in ultrafiltration of coolant lubricant emulsion due to the highest pure water and coolant lubricant fluxes (1146 and 32 L/(m^2^ h), respectively), rejection coefficient (100%), and flux recovery ratio (84%). Furthermore, this membrane featured improved ability of surface contamination degradation after UV irradiation in prolonged ultrafiltration of BSA, demonstrating a high flux recovery ratio (89–94%).

## 1. Introduction

Nowadays, the surface of our planet consists of more than 70% water, but only 6% is fresh water [1]. Oil is the main pollutant of water resources due to rapid industrial development and energy demand growth, which affect living organisms and lead to environmental degradation and pollution [2]. Thus, water purification and its recovery from pollutions, especially from oils, is an important task that needs to be urgently solved. There are various technologies used for water purification, but they become ineffective at low oil concentrations in water [3]. The application of membrane technologies (in particular, ultrafiltration) is a relevant alternative due to efficiency, flexibility, productivity, selectivity, and environmental friendliness [4]. However, porous membranes used in ultrafiltration have an inherent problem of fouling, which increases cleaning costs, equipment breakdown, membrane rupture, and short service life [5]. The introduction of various particles with antibacterial and photocatalytic properties in membranes has attracted great attention for water purification, yielding the reduction of the membrane fouling degree [6,7]. Moreover, this membrane modification provides improved mechanical, optical, electrical, thermal, and chemical stability [8,9,10].

Titanium oxide (TiO_2_) is one of the widespread, commercially available, inexpensive, and promising photocatalysts with excellent chemical stability and high photocatalytic potential [1]. Its use as an independent catalyst has led to concerns about its negative impact on the environment. However, its inclusion in small concentrations in a polymeric material (membrane) may both reduce environmental hazards and improve the separation performance and efficiency of the membrane process for water purification [11,12,13].

In this work, TiO_2_ was introduced into the poly(m-phenylene isophthalamide) (PA) matrix, which is widely used to develop membranes for nanofiltration [14,15], ultrafiltration [16,17], distillation [18], and reverse osmosis [19,20], as well as for the design of tissue biostructures, batteries, air filters, and in electric elements [21,22,23,24,25]. The active application of PA is associated with its unique properties such as mechanical and chemical resistance, commercial availability and low cost, ease of processing, and the possibility to design highly porous materials [26].

There are only a few works devoted to the application of composite polyamide/TiO_2_ in membrane technology, particularly for the preparation of membranes for nanofiltration [27,28,29,30,31,32], reverse osmosis [33,34,35,36,37,38], pervaporation [39], and microfiltration [40]. In [27,28,29,30], the nanofiltration thin film nanocomposite (TFN) membranes based on PA were subjected to the modification with TiO_2_. The TFN PA membrane modified by TiO_2_ was developed for improved salt rejection [27]. The optimal membrane demonstrated enhanced separation performance (in 12% and 19% of water permeability and NaCl rejection, respectively) and antifouling properties with high flux recovery (94%) compared to a pristine PA TFN membrane. In [28], graphene oxide decorated with TiO_2_ nanoparticles was incorporated into the TFN membrane for the efficient organic solvent nanofiltration to achieve improved antifouling properties. The GO nanosheets promoted solvent channels and TiO_2_ provided super surface hydrophilic properties resulting in the formation of a permeable hydrophilic membrane with enhanced antifouling properties. The comparison of the effect of TiO_2_ (15 nm) and SiO_2_ (10–20 nm) as nanofillers in the TFN PA membrane in terms of flux and rejection of organic matter (humic and tannic acids) and salts (MgSO_4_ and NaCl) was carried out in [29]. The permeability of modified PA membranes was increased by 24 and 58% with the incorporation of TiO_2_ and SiO_2_, respectively. The modification of the PA membrane with TiO_2_ significantly improved the MgSO_4_ rejection. However, TiO_2_- and SiO_2_-modified membranes had a lower flux decline ratio (FDR) in organic matter filtration compared to the pristine membrane. TFN PA membranes were prepared by interfacial polymerization on a polyethersulfone (PES) substrate with the incorporation of aminated titanium dioxide (APTES-TiO_2_) in the work [30]. The membrane with 0.3 *w*/*v* % APTES-TiO_2_ had greatly improved pure water flux with a high level of Na_2_SO_4_ rejection (>95%), and NaCl and Na_2_SO_4_ rejection (23.2%, 99.7%, respectively) in the separation of single salt solution. The works in [31,32] were aimed at modifying commercial nanofiltration PA membranes. A commercial PA membrane was modified by coating with silver-doped TiO_2_ (Ag–TiO_2_) nanoparticles (0.05, 0.1, and 0.5 wt.%) on the membrane surface by a dip coating method [32]. The transport properties of the modified membrane were tested in filtration of pure water, saltwater, and by examining the bacterial growth of Gram-positive (*Bacillus subtilis*) and Gram-negative (*Escherichia coli*) on the membrane surface. It was indicated that the modified membranes demonstrated efficient antibacterial properties: the bacterial growth reduced approximately 93% and 91% compared to the pristine membrane. The other commercial PA membrane was surface modified by the coating of nanomaterials graphene oxide (GO) and titanium dioxide (TiO_2_) to enhance membrane separation and antifouling properties for the removal of methylene blue (MB) in water [31]. The increased flux from 28% to 61% and improved antifouling property of the coated membranes (especially under UV-irradiation) were reported.

For reverse osmosis (RO), the modification with TiO_2_ of PA membrane was carried out by various approaches: the introduction of fillers into polymer matrix [33] or the coating of fillers on the surface [34,35,36,37,38]. In [33], a RO PA membrane was developed by the incorporation of GO and TiO_2_ to enhance the salt rejection and filtration performance for purification of drinking water and treatment of wastewater. The introduction of TiO_2_-GO into the membrane matrix improved the membrane performance (62 L/(m^2^ h) flux, 97% salt rejection, and 100% hydrocarbons rejection) due to changes in charge, roughness, and hydrophilicity of the membrane surface. The surface modifification of a commercial RO TFN PA membrane was carried out using irradiated chitosan/titanium dioxide (CS/TiO_2_); the highest flux was demonstrated for the membrane with TiO_2_ concentration of 0.125 wt.% [34]. For RO desalination, a TFN PA membrane was also fabricated with deposition of positively and negatively charged titania nanosheets on the surface by layer-by-layer (LbL) assembly to enhance hydrophilicity [35]. The modification significantly increased water permeability and salt rejection due to the hydration layer hindered the direct contact of salt with the membrane surface. The highest water permeability (0.8 L/(m^2^ h bar)), 60% higher compared to the pristine membrane) and higher NaCl rejection (98.45%) were demonstrated for the membrane with two bilayers. In [36], a RO PA membrane was surface modified by coating with TiO_2_ nanoparticles (10, 20, 30 and 50 ppm) on the membrane and using UV-initiated graft polymerization at different irradiation times and various acrylamide concentrations. The TiO_2_-coated membranes demonstrated the increasing water flux with the increase of nanoparticles concentration and irradiation time, and slightly decreased membrane rejection at low irradiation times. RO membrane modified with both acrylamide and TiO_2_ under UV irradiation had enhanced water flux up to 18% with slightly higher rejection and better antifouling properties compared to the pristine membrane. RO TFN PA membrane was also modified by the layer-by-layer (LbL) assembly of positively and negatively charged titanium nanosheets (TNS) on the surface to improve the fouling resistance for high concentration oily saline water and to maintain a high level of salt rejection [37]. It was demonstrated that the modification improved surface hydrophilicity, roughness, and PA cross-linking. The membrane coated with two bilayers of TNS had an improved permeability, much lower fouling propensity, and improved oil and salt rejection (>99% and >98%, respectively). Additionally, TiO_2_ was deposited on a porous PES support as a sublayer for the fabrication of high-performance RO TFN PA membrane; this increased the affinity between modified PES and amine monomer during the interfacial polymerization [38]. The obtained modified thin film composite (TFC) membrane with TiO_2_ coverage demonstrated improved RO performance (high permeance of 1.8 L/(m^2^ h bar) and salt rejection of 96.1%).

TFN PA membranes were also fabricated by interfacial polymerization on the surface of TiO_2_ modified ceramic hollow fiber (CHF) substrate to improve the pervaporation dehydration performance [39]. The modified TFC membrane had the highest separation index (PSI, 7.83 × 10^7^), separation factor (above 12,000), and the permeation flux (~6.44 kg/(m^2^ h)) in dehydration of 90 wt.% isopropanol at 60 °C. In [40], the modification of a microfiltration PA membrane by TiO_2_ + AgO coating was carried out. The TiO_2_ + AgO coating improved and formed novel functional properties: bactericidal and photocatalytic. The photocatalytic properties of the PA membrane were tested on MB degradation under UV irradiation and visible light. The filtration results showed that membranes covered with two-component TiO_2_ + AgO had a permeate flux similar to the non-coated membrane. The antibacterial properties of TiO_2_ + AgO coatings were evaluated for two bacteria (*Escherichia coli* and *Bacillus subtilis*). The TiO_2_ + AgO coatings on the PA membrane caused a complete suppression of bacterial growth, due to the presence of Ag/AgO nanoparticles and created very good photocatalytic properties due to TiO_2_. However, to the best of our knowledge, the development of composite poly(m-phenylene isophthalamide)/TiO_2_ membranes for ultrafiltration has not been reported so far.

As the development of ultrafiltration membrane with good performance and low surface fouling is a challenging task and is very important for membrane technology, the aim of the present work is to develop and characterize the ultrafiltration membranes based on poly(m-phenylene isophthalamide) with improved transport and antifouling properties related to the membrane modification with TiO_2_ particles. The effect of the introduction of TiO_2_ particles into the PA casting solution on the structure, separation performance, and antifouling stability of PA porous ultrafiltration membranes was thoroughly studied in this work. The transport properties were studied in ultrafiltration of industrially important feeds: coolant lubricant (cutting fluid) emulsion and bovine serum albumin (BSA) solution. The characterization of membrane structure and physicochemical properties was carried out by different methods: scanning electron and atomic force microscopies, the measurements of contact angle, and total porosimetry. Moreover, the cleaning of surface contamination of modified membranes after the ultrafiltration was carried out using the UV lamp illumination; the flux recovery ratio was also evaluated.

## 2. Materials and Methods

### 2.1. Materials

**Figure 1:**
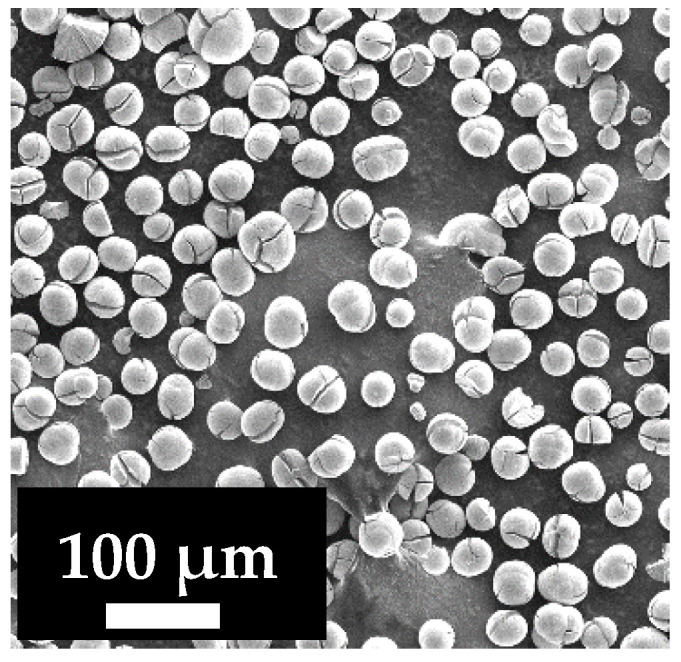
The SEM micrograph of TiO_2_ particles used for membrane modifications.

Poly(m-phenylene isophtalamide) (PA, Fenylon C2, lot. 146/19, “UNIPLAST” Ltd., Vladimir, Russia) was used as a membrane material. Titanium oxide (TiO_2_, >99.9%, PKF “Non-ferrous metallurgy” Ltd., Yekaterinburg, Russia) was used for the modification of porous PA membranes. The average particle size of TiO_2_ was determined by scanning electron microscopy (SEM) (Figure 1) and equals ~20 µm. *N*,*N*′-Dimethylacetamide (DMA) and lithium chloride LiCl (“Vekton”, St. Petersburg, Russia) were used without further treatment. Coolant lubricant (cutting fluid) (Wittol 297, SERVOVIT, Minsk, Belarus) emulsion (5 wt.% in water) and 0.5 wt.% bovine serum albumin (BSA, Mw = 67,000 g/mol, “Sigma-Aldrich”, St. Louis, MO, USA) solution in phosphate buffer (pH 7.0–7.2) were applied to evaluate membrane separation performance and antifouling properties.

### 2.2. Porous Membrane Preparation

Anisotropic porous membranes were prepared as follows: a predetermined amount of PA powder was dissolved in DMA with 0.66 wt.% lithium chloride at 100 °C with a constant stirring for 3 h to obtain 12 wt.% polymer solution. Porous PA membranes were prepared by phase inversion technique: the PA solution gradually cooled to ambient temperature (25 °C) was cast with a casting blade (200 µm gap width) onto a glass plate and then immersed in a coagulation bath with distilled water (non-solvent induced phase separation (NIPS)). The thickness of the prepared porous PA membranes measured by the micrometer was 80 ± 10 μm.

The modification of porous PA membranes using TiO_2_ was carried out by grinding the PA powder with the predetermined amount of TiO_2_ particles (0.3, 0.5, 0.75 wt.% with respect to the polymer weight) in an agate mortar with a further dissolution of composites in DMA with LiCl as described above. The formation of porous membranes based on a composite PA/TiO_2_ was carried out in the same way as for a membrane based on a pristine polymer described above. The thickness of the porous PA/TiO_2_ membranes measured by the micrometer was equal to 90 ± 10 μm, which does not differ much from the thickness of the membrane based on pristine PA and not significantly affect the transport properties of the modified membranes.

**Figure 2:**
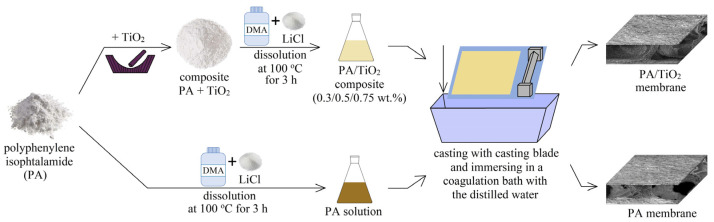
The scheme of preparation of porous membranes based on PA and its composites with TiO_2_.

The preparation of porous membranes based on PA and its composites with TiO_2_ is presented schematically in Figure 2.

### 2.3. Structural Investigation of the Membranes

The inner and surface structure of porous membranes was investigated by scanning electron (SEM) and atomic force (AFM) microscopies, the standard porosimetry method, and contact angle measurements. The inner morphology of the developed porous PA-based membranes was investigated with a Phenom Pro scanning electron microscope (Thermo Fisher Scientific Inc., Waltham, MA, USA). Membranes were cleaved in liquid nitrogen followed by the application of a gold layer using a cathode sputtering vacuum installation DSR (Vaccoat, London, UK). The surface of the porous PA-based membranes was investigated with an NT-MDT NTegra Maximus atomic force microscope (“NT-MDT Spectrum Instruments”, Moscow, Russia) in the tapping mode. The pores in the PA-based membranes were measured with a Porosimeter 3.1 instrument (POROTECH Ltd., Woodbridge, ON, Canada) by the standard porosimetry method; n-octane was applied as a reference liquid. Water contact angle of the porous PA-based membranes was measured by the attached bubble method with a Goniometer LK-1 device (“NPK Open Science” Ltd., Krasnogorsk, Russia) to assess the hydrophilic surface properties [41]. To analyze contact angle data, the “DropShape” software was applied. For each membrane, at least three different locations were tested, and the average contact angle values were calculated.

### 2.4. Determination of Separation and Antifouling Membrane Performance

**Figure 3:**
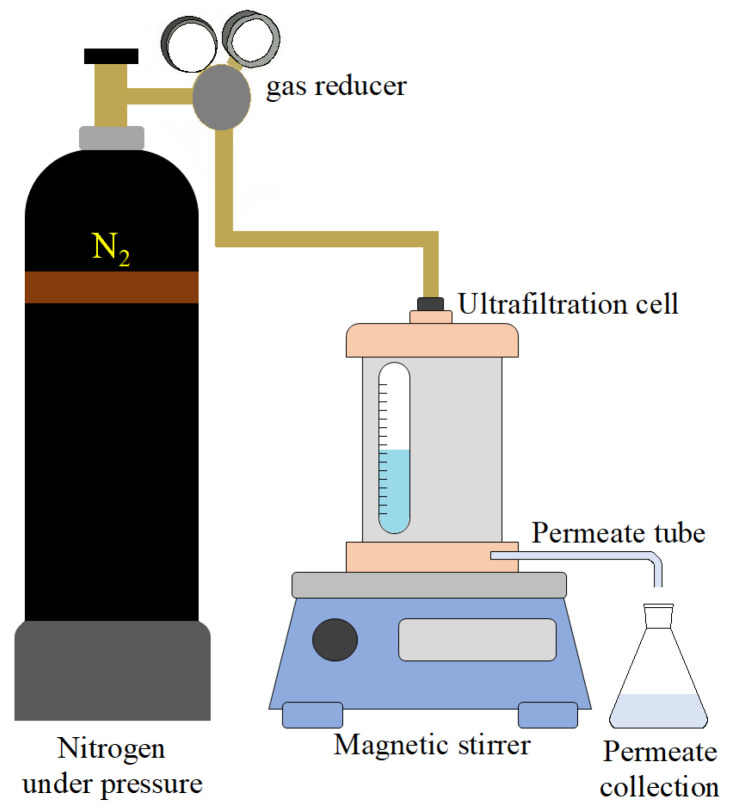
The scheme of ultrafiltration set-up.

Transport properties of the developed PA-based membranes were evaluated by ultrafiltration in a stirred filtration Amicon-type setup with an effective membrane area of 24.6 cm^2^ (Figure 3). Pure water flux, feed flux, and rejection coefficient were determined in stirred ultrafiltration at the transmembrane pressure of 1 bar and ambient temperature (25 °C) at a stirrer rate of 300 rpm. Emulsions of coolant lubricant in water (5 wt.%) and 0.5 wt.% BSA solution in phosphate buffer were used as feed solutions in ultrafiltration experiments. First, the membrane was pre-conditioned upon ultrafiltration of distilled water for 30 min at a transmembrane pressure of 1 bar. Thereafter, the pure water flux was determined according to Equation (1). Then, the water was replaced with the feed solution in the ultrafiltration cell. The permeate flux and rejection coefficient were determined after 10 min of filtration according to Equations (1) and (2), respectively.

The flux (*J*) of membranes was calculated as follows [42]:

(1) J=VA×t,

where *A* is the effective membrane area (m^2^), *V* is the volume of permeate (L), and *t* is the permeation time (h).

The content of coolant lubricant and BSA in permeates was investigated by spectrophotometry using a Spectrophotometer PE-5400UV (“ECROCHEM”, Moscow, Russia) at a wavelength of 500 and 280 nm corresponding to the absorbance maximum for the coolant lubricant and BSA, respectively.

The rejection coefficient (R, %) was calculated as follows:

(2) R=(1−CpCf)×100%,

where *Cp* and *Cf* are the foulant contents in the permeate and the feed (g/L), respectively.

For evaluation of membrane transport properties, at least three different membrane samples were tested, and the average flux and rejection coefficient were calculated. The relative error was found to be less than 5% for the flux measurements and less than 0.5% for the rejection coefficient.

To study membrane antifouling performance, the following procedure was applied. The membrane was pre-conditioned for 30 min at 1 bar and ambient temperature (25 °C). Then, the pure water flux was determined. The feed BSA solution or coolant lubricant emulsion was placed in the ultrafiltration cell and filtered at 1 bar for 40 min. The flux was measured every 10 min. Thereafter, the pure water flux of the membrane after the BSA solution or coolant lubricant aqueous emulsion ultrafiltration was determined. The flux recovery ratio (FRR) was calculated as follows:

(3) FRR=(JJ0)×100%,

where *J* is the flux of pure water after the contact of the membrane with foulant and *J_o_* is the initial pure water flux [43].

To evaluate the cleaning ability of the modified PA/TiO_2_ membranes and reference PA membrane, the contaminated with BSA membrane after rinsing with water in ultrafiltration setup was immersed into Petri dishes with water under an ultraviolet DRT-125 lamp (230–400 nm, MEDtechnique No. 7, St. Petersburg, Russia) for 2.5 h. After the membrane was taken out, its pure water flux was measured again in order to consider the flux recovery ratio according to Equation (3) [44]. To investigate long-term membrane stability, the PA and PA/TiO_2_ (0.3) membranes were tested in ultrafiltration of BSA solution with ultraviolet illumination for 2.5 h during four cycles for two days. The membrane flux was measured every 10 min. Two cycles were carried out during one day, then after the second UV irradiation, the membranes were left immersed in water in Petri dishes for a night and tested again the next day.

## 3. Results and Discussions

### 3.1. Characterization of PA and PA/TiO_2_ Membranes

**Figure 4:**
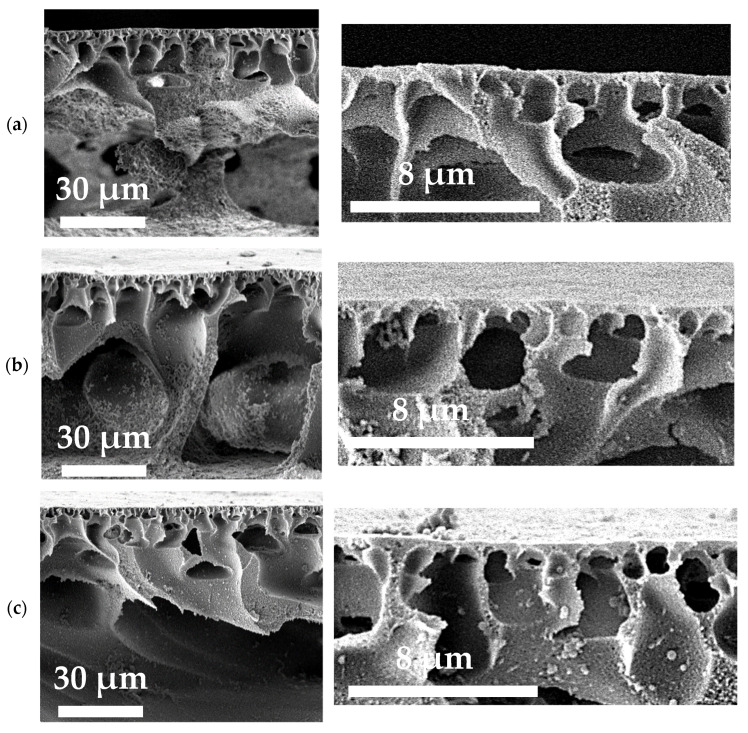

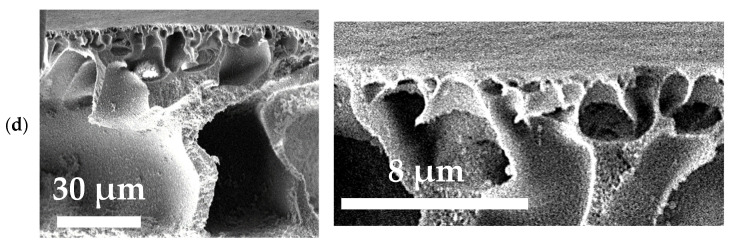
SEM cross-sectional micrographs of the membranes based on PA (**a**) and PA/TiO_2_ (0.3 (**b**), 0.5 (**c**), and 0.75 (**d**) wt.%) composites.

The effect of TiO_2_ particles incorporation in the PA membrane matrix on the membrane structure was studied using SEM and AFM. The membrane structure is known to determine membrane transport properties as well as membrane antifouling performance. To explain the change of membrane transport properties in ultrafiltration due to modification by TiO_2_ particles, the structure of the membrane cross section and surface of the selective layer of PA and PA/TiO_2_ membranes was investigated. The cross-sectional SEM micrographs of the porous membranes are presented at different scales in Figure 4.

**Figure 5:**
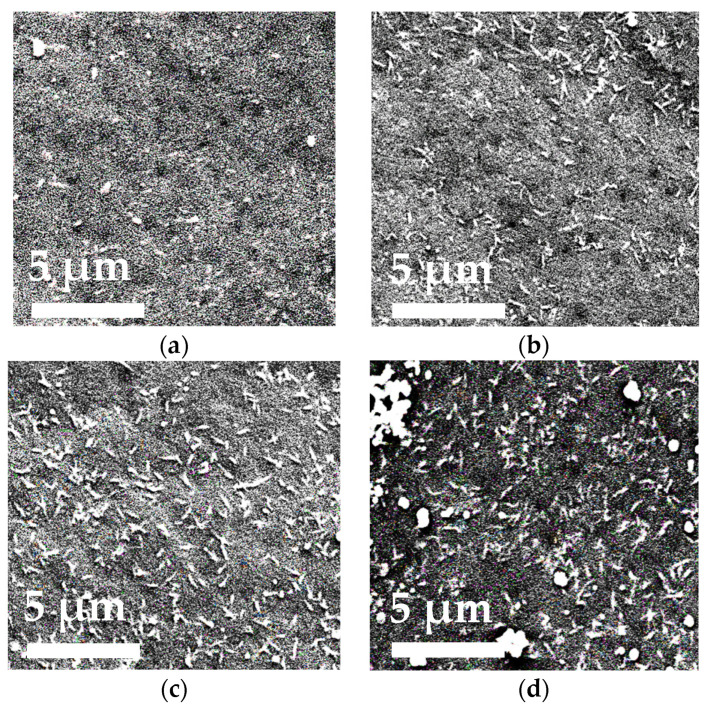
SEM images of the surface of the selective layer of membranes based on PA (**a**) and PA/TiO_2_ (0.3 (**b**), 0.5 (**c**), and 0.75 (**d**) wt.%) composites.

It was found that all prepared membranes featured asymmetric structure of the cross section with thin selective layer and porous membrane matrix. The porous membrane matrix is pierced by the large cellular macrovoids, typical of membranes prepared by non-solvent induced phase separation. It was found that the narrow macrovoids passed into wider and larger macrovoids of the PA membrane further from the membrane selective layer compared to the PA/TiO_2_ membranes, where the broad macrovoids were located much closer to the selective layer. The increase in TiO_2_ concentration from 0.3 to 0.75 wt.% in the PA matrix increased macrovoid size. The selective layer became less dense and more porous, contributing to the increase in the flux of the TiO_2_-modified membranes in comparison to the membrane based on pristine PA. The effect of TiO_2_ incorporation into the membrane matrix on the membrane transport properties will be discussed in detail in Section 3.2. The SEM images of the surface of the selective layer are presented in Figure 5.

**Figure 6:**
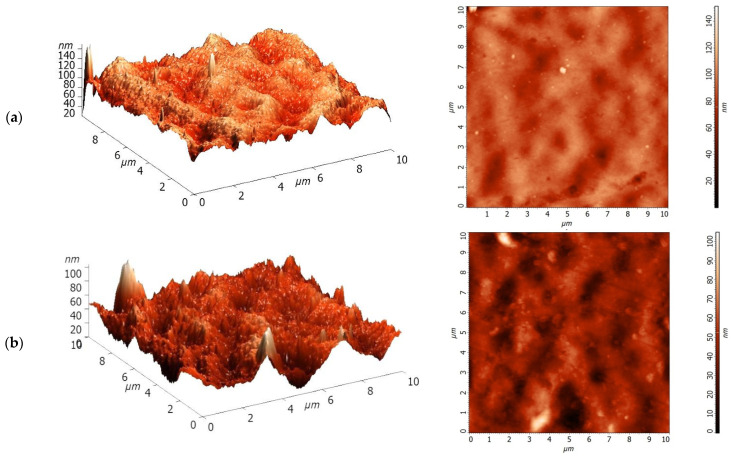

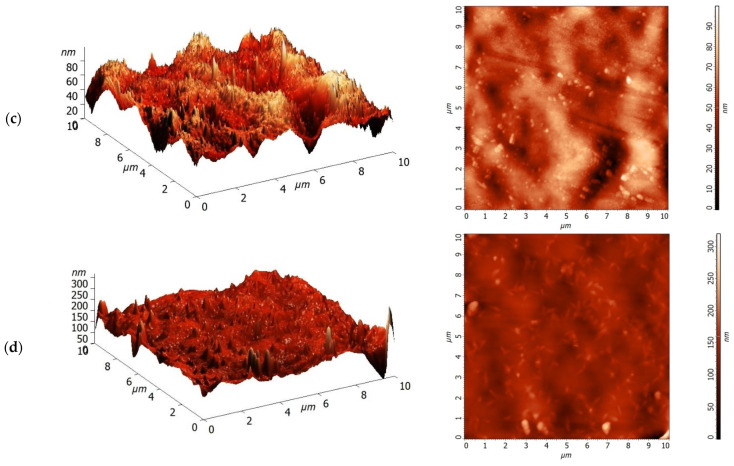
AFM images of the membranes based on PA (**a**) and PA/TiO_2_ (0.3 (**b**), 0.5 (**c**), and 0.75 (**d**) wt.%) composites.

The modification of the PA membrane with TiO_2_ led to the accumulation of particles and their aggregates on the surface of the membrane selective layer. The same trend of TiO_2_ particles aggregates on polybenzimidazole (PBI) membranes was also reported in the work [45]. It was found that the size and the number of TiO_2_ particles and their aggregates significantly increased with the increase of the modifier content in the membrane matrix. The surface of the selective layer was found to be rather rough, featuring cavities which may contain pores at their bottom. These cavities may be attributed to the pore openings. However, their size, which was determined using the SEM images, was too large for ultrafiltration membranes taking into account the rejection of the developed membranes, which will be discussed in detail in Section 3.2. The membrane separation performance (Section 3.2) indicates that the size of the transport pores is much smaller compared to the size of these cavities observed on the surface of the membrane selective layer. Note that SEM is not a perfect tool for the study of the porous structure of the membrane selective layer as it gives the opportunity to observe the shape and size of the pore only on the surface of the membrane selective layer. However, the pore can change its size along the thickness of the selective layer which cannot be monitored by SEM, so the size of transport pores can be inconsistent with those observed on the surface of the selective layer by SEM. The quantity and density of cavities of the PA membrane compared to the PA/TiO_2_ membranes is smaller. However, at TiO_2_ concentration over 0.3 wt.%, the particles began to clog these surface cavities. This may change membrane surface structure significantly affecting the membrane antifouling properties. In addition, TiO_2_ particles on the surface of the modified membranes play an important role in determining the hydrophilic and photocatalytic properties discussed in the following sections. The changes in surface topography of the membranes were studied by AFM. AFM images of the PA and PA/TiO_2_ membranes are presented in Figure 6.

It was revealed that the surface of the selective layer featured the nodule structure typical for membranes prepared by non-solvent-induced phase separation. Similar to the SEM images, AFM studies revealed that the introduction of TiO_2_ particles into the membrane matrix resulted in the formation of more pores (cavities) on the surface of the selective layer. Moreover, deeper cavities with sharper edges were formed for the PA/TiO_2_ composite membranes compared to the reference PA membrane (Figure 6). Large aggregates of TiO_2_ particles are observed on the selective layer surface for the PA/TiO_2_ membranes. An increase in TiO_2_ concentration with respect to the PA weight increases the number of TiO_2_ particle aggregates on the membrane surface.

To evaluate membrane surface roughness during modification, surface parameters of the PA and PA/TiO_2_ membranes were calculated based on AFM images in terms of the root mean squared surface roughness (Rq) and average roughness (Ra) (Table 1). Water contact angle determined by the attached bubble method and total porosity data for the PA and PA/TiO_2_ membranes are presented in Table 1.

The accumulation and aggregation of TiO_2_ particles on the membrane surface (confirmed by SEM and AFM data, Figure 5 and Figure 6) increased the surface roughness parameters of the modified membranes compared to the non-modified PA membrane, which increased with the rise of TiO_2_ content in the PA matrix (Table 1). The increased surface roughness of modified membranes would significantly affect contact between foulants (coolant lubricant and BSA) and membrane increasing the tendency to membrane fouling [44]. The total porosity data obtained by the standard porosimetry method also indicate that an increase in the modifier content above 0.3 wt.% partly clogs the membrane pores. The porosity of the PA/TiO_2_ (0.5 and 0.75 wt.%) membranes decreases slightly to 83 and 79%, respectively, compared to the membranes based on the pristine PA (88%) and PA/TiO_2_ (0.3 wt.%) composite (90%). The water contact angle data indicate the surface hydrophilization of the PA membrane during the modification with TiO_2_ [46]. The water contact angle significantly decreases with an increase in the TiO_2_ concentration in the PA matrix, contributing to the changes in the separation and antifouling properties of the membranes, which will be discussed in Section 3.2. Water molecules are adsorbed on the membrane surface due to the TiO_2_, forming a thin boundary on the membrane surface and increasing its hydrophilicity, which promotes more intensive penetration of water molecules through the membrane, increasing the flux and reducing the degree of membrane fouling [32].

The results of the structural studies of the membranes indicate that TiO_2_ introduction into the membrane matrix affects the mechanism of non-solvent phase separation. Hydrophilic TiO_2_ particles migrate to the membrane surface in NIPS due to the affinity to the non-solvent (water) and can partly be washed out to the coagulation bath together with the pore former (LiCl). It results in a significant change of pore number and size in the selective layer. Moreover, the migration of TiO_2_ particles to the membrane surface leads to the deposition of TiO_2_ particle aggregates on the membrane surface, which changes surface roughness and water contact angle of the selective layer.

### 3.2. Transport Properties of PA and PA/TiO_2_ Membranes in Ultrafiltration

#### 3.2.1. Ultrafiltration of Coolant Lubricant Emulsion

**Figure 7:**
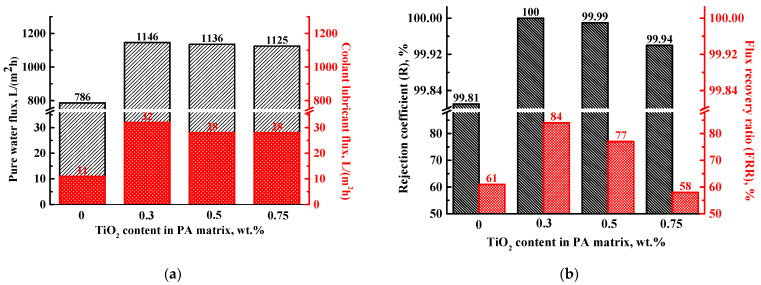
The dependence of (**a**) pure water and coolant lubricant fluxes, (**b**) rejection coefficient (R), and flux recovery ratio (FRR) on the TiO_2_ content in the PA membranes.

The increase in energy demand leads to the rise of oil and gas exploration and production generating large volumes of oily wastewater. Various technologies are applied to treat this water, but their effectiveness decreases with decreasing oil content, and the fraction of dissolved oil in water can be retained [3]. Membrane separation by ultrafiltration is one of the high-performance, selective, and efficient technologies for removing oil from water. Thus, the developed porous PA-based membranes were tested in ultrafiltration with a coolant lubricant (cutting fluid) emulsion (5 wt.% in water) to assess their promising application in industry. Transport properties of the PA and PA/TiO_2_ membranes are presented in Figure 7.

The ultrafiltration data demonstrate that the modification of the PA membrane by TiO_2_ improves pure water and coolant lubricant emulsion fluxes (Figure 7a) of modified membranes, which may be due to the surface hydrophilization (Table 1) [45,46] and the changes in the structure of the selective layer (less dense and more porous selective layer, Figure 4). The obtained data are also confirmed by the previous research, where it was shown that the addition of hydrophilic particles into the polymer matrix increased the stratification during membrane preparation due to increased thermodynamic instability [47], which resulted in membrane with higher porosity, pore radius, and surface porosity. This indicates that the addition of TiO_2_ in PA modified the pore system of the membrane and changed its hydrophilicity and pore structure, causing an increase in membrane pure water and water solution permeability [29].

Note that all membranes had a high rejection coefficient of coolant lubricant (from 99.81 up to100 wt.% in the permeate) (Figure 7b). The modified membranes have slightly higher coolant lubricant rejection compared to the PA membrane (99.81 wt.%). It can be explained by the fact that the introduction of TiO_2_ into the PA matrix probably blocked some of the pores of the membrane (confirmed by SEM and total porosity data, Figure 5 and Table 1) resulting in a rejection improvement [48]. However, the best flux recovery ratio was found for the PA/TiO_2_ (0.3 wt.%) membrane (84%) compared to membranes based on the pristine PA (61%) and PA/TiO_2_ (0.5 and 0.75 wt.%) composites (Figure 7b). The FRR value depends on the hydrophilicity, pore size, and roughness of the selective membrane layer. The increased surface hydrophilicity of the filled membranes should reduce membrane fouling (increase in FRR). As membrane permeability (and thus surface pore size) increases compared to the pristine PA membrane, it may promote increased availability of the selective layer structure for the pollutant (coolant lubricant) facilitating its penetration inside the pores [49]. However, the optimal content of TiO_2_ (0.3%) in the PA matrix forms a balance between surface hydrophilicity, roughness, and pore size of the membrane, maintaining high performance (fluxes) and FRR [29]. Despite the increase in surface hydrophilicity of the PA/TiO_2_ (0.5 and 0.75 wt.%) membranes, their higher surface roughness compared to the PA and PA/TiO_2_ (0.3 wt.%) membranes (Table 1) caused an increase in the specific area of the selective layer for the adsorption of coolant lubricant molecules. This results in higher contamination by pollutants and a decrease in FRR compared to the PA/TiO_2_ (0.3 wt.%) membrane [50].

Moreover, the increased FRR of the PA/TiO_2_ (0.3 wt.%) membrane compared to the PA membrane is related to the significant surface hydrophilization (decrease of water contact angle), which counterbalances a slight increase in surface roughness. The PA/TiO_2_ (0.3 wt.%) membrane demonstrates better antifouling performance compared to the other modified membranes due to the absence of strong TiO_2_ agglomeration on the surface of the selective layer. All these factors resulted in improved flux, R, and FRR due to simplified penetration of water molecules through the unblocked and more hydrophilic pores. Thus, the optimal ultrafiltration properties were obtained for the PA/TiO_2_ (0.3 wt.%) membrane, which was further studied for photocatalytic activity.

#### 3.2.2. Ultrafiltration of BSA Solution and Flux Recovery of Membranes by Photocatalysis

**Figure 8:**
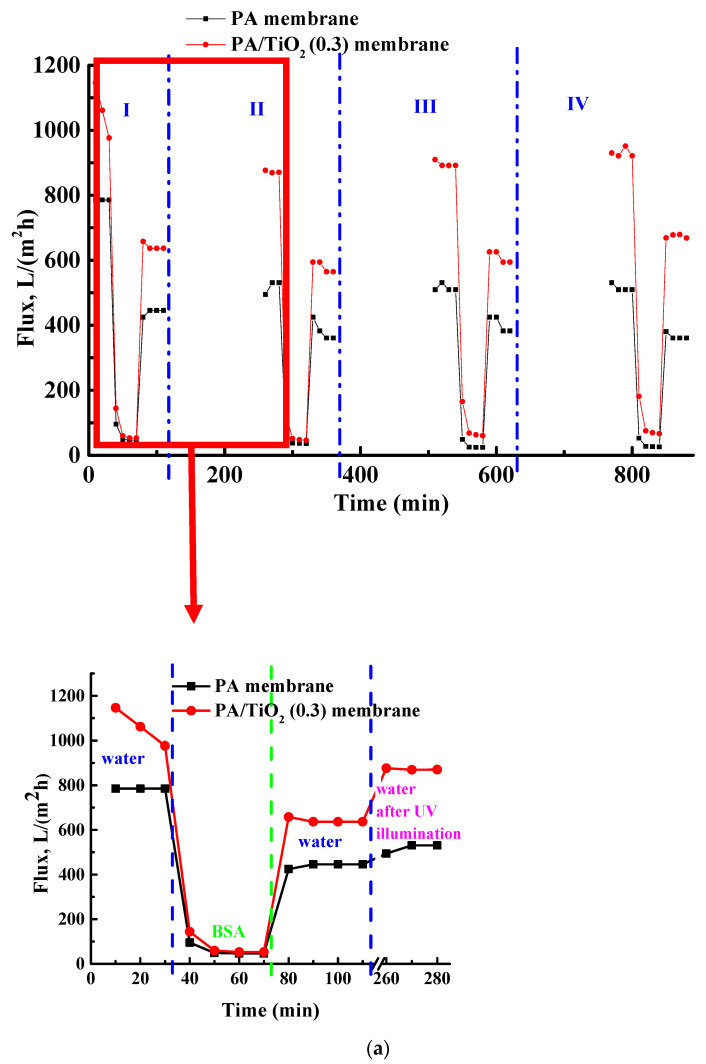

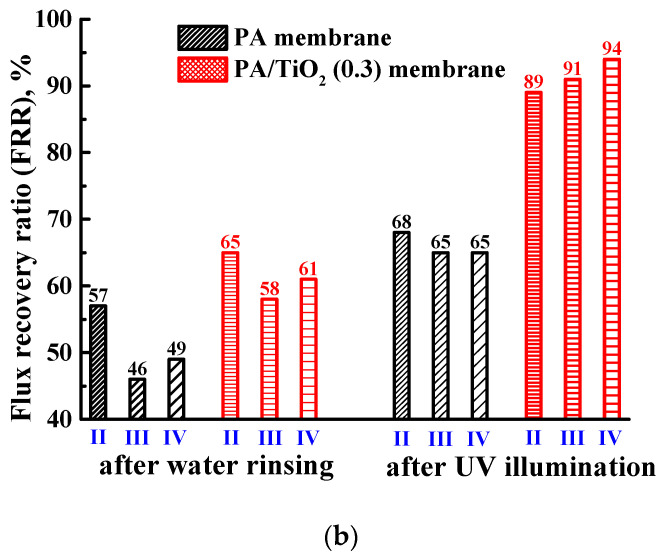
Performance and antifouling stability of the reference PA and modified PA/TiO_2_ (0.3) membranes in ultrafiltration of BSA solution: (**a**) the dependence of flux on the time of ultrafiltration; (**b**) flux recovery ratio (FRR) after water rinsing and after ultraviolet illumination for 2.5 h during four cycles. Rejection coefficient of BSA for all membranes was ~60 ± 5%.

The designed porous PA/TiO_2_ (0.3 wt.%) membrane was tested in prolonged ultrafiltration sessions using BSA as a model protein to study the cleaning ability under lamp illumination after the reversible fouling. The obtained data are presented in Figure 8. The membrane based on pristine PA was also investigated in prolonged ultrafiltration for the comparison with the modified membrane to evaluate the changes during the modification with TiO_2_.

To investigate the long-term membrane stability, the PA and modified PA/TiO_2_ (0.3) membranes were tested in ultrafiltration of BSA solution with ultraviolet illumination during four cycles (for two days) (Figure 8a). It was demonstrated that the rejection coefficient of BSA for both membranes was similar and equal to ~60 ± 5% during the whole experiment. Pure water flux of the PA membrane had the constant values during 30 min prefiltration (785 L/(m^2^ h)), while it was slightly decreased for the PA/TiO_2_ (0.3 wt.%) membrane (from 1146 to 977 L/(m^2^ h)). During the BSA filtration process, the flux of the PA and PA/TiO_2_ (0.3 wt.%) membranes decreased sharply to 47 and 53 L/(m^2^ h) (Figure 8a), respectively, which could be explained by the BSA deposition on the membrane surface causing the formation of BSA gel layers [44]. After the water flushing of the membranes for 40 min, the pure water flux of the PA and PA/TiO_2_ (0.3 wt.%) membranes recovered by 57% and 65% of the initial pure water flux value after 30 min of pre-filtration, respectively (Figure 8b). Afterwards, the membranes were immersed in water under UV lamp illumination for 2.5 h to evaluate the photocatalytic activity. After this, the flux of the PA membrane recovered by 68%, and the flux of the PA/TiO_2_ (0.3 wt.%) membrane to 89% (Figure 8b). Further, the same three cycles were repeated for the membranes (Figure 8a). FRR after the UV illumination for the PA/TiO_2_ (0.3 wt.%) membrane increased from 89% to 94% during four cycles, while for the PA membrane R slightly decreased from 68% to 65% and was significantly lower compared to the modified membrane (Figure 8b). The lower FRR values after water rinsing for both membranes (Figure 8b) compared to FRR values after the UV illumination indicated the effectiveness of UV application.

The improved recoverability of the modified membrane flux indicates the photocatalytic activity: titanium oxide in the PA matrix absorbing a quantum of light generates free charge carriers: negative electrons and positive vacancies (holes) [51]. They react with oxygen and water vapor by oxidation-reduction reactions forming strong oxidizing agents (O_2_−, −OH, and radicals). Active species directly interact, degrade, and mineralize organic pollutants on the membrane surface, increasing in the removal degree of impurities from the membrane [52]. The high values of R, FRR, and similar values of pure water and protein fluxes of the modified PA/TiO_2_ (0.3 wt.%) membrane and their constancy show its effectiveness in long-term filtration experiments and the indelibility or washout of titanium oxide particles from the polymer matrix. Thus, the modified PA/TiO_2_ (0.3 wt.%) membrane has a higher flux and due to its photocatalytic properties exhibits self-cleaning properties after UV illumination, when contaminants on the membrane surface and in the pores can be effectively removed providing a high level of flux recovery ratio.

## 4. Conclusions

In this study, the improvement of transport properties and antifouling stability of the ultrafiltration poly(m-phenylene isophthalamide) (PA) membranes was achieved by modification with TiO_2_ particles. Thus, the novel ultrafiltration poly(m-phenylene isophthalamide) (PA)/TiO_2_ membranes were prepared by non-solvent-induced phase separation. The analysis of transport characteristics of membranes was carried out in ultrafiltration of industrially relevant feeds: coolant lubricant (cutting fluid) emulsion in water and bovine serum albumin solution. Antifouling performance was studied in prolonged ultrafiltration using BSA solution.

The investigation of membranes in ultrafiltration of coolant lubricant (cutting fluid) emulsion demonstrated that the modification of PA membrane by TiO_2_ improved pure water and coolant lubricant fluxes of modified membranes, which could be related to the surface hydrophilization (confirmed by water contact angle measurements) and the formation of less dense and more porous selective layer (confirmed by SEM data). Furthermore, note that all membranes had a high rejection coefficient of coolant lubricant (from 99.81 up to 100 wt.% in the permeate). Moreover, the modified membranes had slightly higher coolant lubricant rejection compared to the PA membrane (99.81 wt.%) because the introduction of TiO_2_ into the PA matrix blocked some of the pores of the membrane (confirmed by SEM and total porosity data). Among all studied membranes, the PA/TiO_2_ (0.3 wt.%) membrane demonstrated the best ultrafiltration properties (FRR 84%, pure water and coolant lubricant fluxes (J) 32 and 1146 L/(m^2^ h), respectively, R 100%) caused by the significant selective layer surface hydrophilization, which counterbalances a slight increase in surface roughness, as well as the absence of strong TiO_2_ agglomeration on the surface in contrast to other modified membranes.

The study of PA/TiO_2_ (0.3 wt.%) membrane separation and antifouling performance in prolonged ultrafiltration using BSA solution was performed to analyze the cleaning ability after reversible fouling under UV lamp illumination. It was demonstrated that the membrane exhibited self-cleaning properties after UV illumination: contaminants on the membrane surface and in the pores were effectively removed providing a high level of flux recovery ratio (89–94%).

## Figures and Tables

**Table 1:** Surface roughness parameters, total porosity, and water contact angle of PA and PA/TiO_2_ membranes.

Membrane	Ra, nm	Rq, nm	Total Porosity, %	Contact Angle, °
PA	7.9	10.1	88	31 ± 2
PA/TiO_2_ (0.3)	8.5	11.0	90	25 ± 2
PA/TiO_2_ (0.5)	9.8	12.6	83	22 ± 2
PA/TiO2 (0.75)	14.0	18.9	79	20 ± 2
